# Translabyrinthine Resection of Posterior Petrous Face Meningiomas: Surgical, Functional, and Oncological Outcomes and Associations with Anatomical Extension and Tumor Burden

**DOI:** 10.3390/cancers18172887

**Published:** 2026-09-06

**Authors:** Ufuk Erginoglu, Taha Sukru Korkmaz, Bekir Can Kendirlioglu, Zeynep Arzum Uyaniker, Umid Sulaimanov, Cagdas Ataoglu, Nafiye Sanlier, Ugur Erginoglu, Eray Tekirdas, Jason Alexander Brant, Joseph Roche, Mustafa Kemal Baskaya

**Affiliations:** 1 Department of Neurological Surgery, University of Wisconsin School of Medicine and Public Health, Madison, WI 53792, USA; erginoglu@wisc.edu (U.E.); t.sukrukorkmaz@gmail.com (T.S.K.); can.kendirlioglu@gmail.com (B.C.K.); arzumuyaniker@gmail.com (Z.A.U.); sulaimanov@wisc.edu (U.S.); drcagdas500@gmail.com (C.A.); nafiyesanlier@gmail.com (N.S.); eraytekirdas@gmail.com (E.T.); 2 Department of Otolaryngology-Head and Neck Surgery, Basaksehir State Hospital, 34480 Istanbul, Turkey; 3 Department of Otolaryngology-Head and Neck Surgery, University of Wisconsin School of Medicine and Public Health, Madison, WI 53792, USA; jabrant@wisc.edu (J.A.B.); jproche@wisc.edu (J.R.)

**Keywords:** anatomical extension, extent of resection, facial nerve outcomes, posterior petrous face meningioma, translabyrinthine approach, tumor burden

## Abstract

**Background/Objectives:** Translabyrinthine (TL) surgery is established for vestibular schwannomas, but its role in a homogeneous posterior petrous face meningioma (PPFM) cohort remains undefined. We characterized surgical, functional, and oncological outcomes and associations with extension and tumor burden. **Methods:** We retrospectively reviewed 14 patients undergoing TL-only resection at a single institution (2006–2024). Petrosal zones (anterior, middle, posterior) were modeled independently. Outcomes included extent of resection, Simpson grade, complications, tumor control, House–Brackmann (HB) facial nerve (FN) grade, modified Rankin Scale (mRS), and Glasgow Outcome Scale (GOS). **Results:** Preoperatively, 10 (71.4%) had non-serviceable or absent hearing, and the median diameter was 33.5 mm. Gross or near-total resection was achieved in 85.7% (median extent, 96.2%). At 1 year, 13 of 14 (92.9%) had HB I–II, including all 12 with favorable preoperative function; all had favorable outcomes (mRS 0–2; GOS 4–5). Over a median 65.5-month follow-up, one recurrence and three regrowths occurred, with five patients (35.7%) receiving adjuvant radiation. In exploratory analyses, anterior involvement was associated with a lower likelihood of Simpson grade 1–2 resection (*p* = 0.025), but not with volumetric extent; tumor diameter correlated with complication burden (ρ = 0.585; *p* = 0.028), and tumor volume correlated with operative duration (ρ = 0.607; *p* = 0.021) and hospital stay (ρ = 0.753; *p* = 0.002). **Conclusions:** In this first TL-only PPFM series, FN and functional outcomes were favorable and tumor control was acceptable under multimodal management. These findings describe outcomes achievable through the TL corridor in appropriately selected patients rather than an advantage over alternative approaches. Exploratory associations between anterior extension, tumor burden, and outcome require confirmation in larger cohorts.

## Simple Summary

Posterior petrous face meningiomas are uncommon, usually benign tumors that grow near the nerves controlling hearing and facial movement. The translabyrinthine approach provides direct access to this region but sacrifices hearing, so it is generally considered when hearing is already poor or unlikely to be preserved. We reviewed 14 patients treated with this approach to describe tumor removal, facial nerve function, complications, and tumor control and to explore whether tumor location and size were related to these outcomes. Most tumors were almost completely removed, facial nerve and overall function were usually favorable at one year, and tumor control was acceptable during follow-up, with adjuvant radiation in some patients. Tumors extending farther forward were less likely to allow complete removal of the involved lining and bone, while larger tumors were linked to greater surgical complexity and more complications. These findings may help surgeons select patients and plan the safest surgical route.

## 1. Introduction

Posterior petrous face meningiomas (PPFMs) are a distinct subgroup of cerebellopontine angle (CPA) tumors arising from the dura of the posterior petrous surface, in close relationship with the internal auditory canal (IAC), cranial nerves V–VIII, and the major venous structures of the posterior fossa [1,2]. Presentation ranges from incidental detection to cranial nerve dysfunction and mass effect, varying with tumor size and anatomical extension [1,3,4]. They lie within the broader, anatomically heterogeneous spectrum of petrous region meningiomas—including petroclival, petrous apex, and petrotentorial lesions—which differ in dural epicenter, neurovascular displacement, and surgical strategy [4,5,6,7].

Although frequently grouped with these lesions in surgical series, PPFMs differ fundamentally in dural origin and neurovascular displacement: they arise lateral to the trigeminal nerve (TN) and characteristically displace the facial–vestibulocochlear complex anteriorly or anteromedially while extending along the posterior petrous surface [3,6], whereas petroclival meningiomas originate medial to the TN. The TN thus serves as a key landmark distinguishing the two, with lateral lesions achieving higher rates of complete resection and lower abducens morbidity than medially situated petroclival meningiomas [8]. These differences carry direct implications for surgical planning, achievable resection, and cranial nerve outcomes.

Accordingly, several skull base corridors have been described for PPFMs—the retrosigmoid, translabyrinthine, anterior transpetrosal (Kawase), and combined transpetrosal approaches, with more recent endoscopic transorbital reports [4,9,10,11,12,13,14]. Corridor choice is determined by tumor location, hearing status, IAC involvement, neurovascular anatomy, and surgeon experience [3,4]. The retrosigmoid approach remains most widely adopted, particularly with serviceable hearing and retromeatal attachment [1,3,5,7,15]. The translabyrinthine (TL) approach, by contrast, provides direct access to the lateral CPA and IAC without cerebellar retraction, allows early facial nerve (FN) identification at the meatal fundus, and exposes the posterior petrous attachment for early devascularization before intradural dissection; because it sacrifices labyrinthine function, it has traditionally been reserved for non-serviceable hearing or cases unlikely to retain hearing on audiologic and anatomical grounds [4,9,10].

Despite its established role in vestibular schwannoma surgery [16], the specific contribution of the TL approach to PPFM management remains incompletely characterized. Existing evidence falls into three categories: heterogeneous multi-approach PPFM series reporting outcomes across corridors selected by anatomy, hearing, and surgeon preference [4,6,17,18]; dedicated single-corridor PPFM series, which to date have relied exclusively on the retrosigmoid approach [1,2,5,15,19,20,21]; and TL-based skull base series that embed PPFMs within anatomically heterogeneous populations alongside petroclival, petrous apex, petrotentorial, and other meningiomas [4,9,10]. As a result, outcomes achievable through a TL approach within a homogeneous PPFM population remain poorly defined, and the influence of the surgical corridor cannot readily be distinguished from that of tumor anatomy on resection, cranial nerve preservation, and morbidity. This gap is clinically consequential because the TL corridor is not selected at random: it is chosen for a recurring and specific situation—hearing that is non-serviceable or unlikely to be preserved, together with fundal or IAC involvement—in which the retrosigmoid corridor offers no auditory advantage. Because TL-treated patients have appeared as subgroups within heterogeneous cohorts rather than as a defined denominator, outcomes attributable to this corridor alone have not been isolated.

PPFMs are anatomically heterogeneous lesions that variably involve the anterior, middle, and posterior petrous surface and may extend into the IAC, factors that may influence resectability and complication risk. Existing classification systems capture this heterogeneity through patterns of dural attachment and extension [1,4,20,22]. A more granular understanding of how these patterns affect outcomes could improve patient selection and operative planning for a TL strategy.

In this single-institution retrospective cohort, we evaluated 14 consecutive patients with imaging and intraoperative findings consistent with PPFMs and histopathologically confirmed meningioma who underwent resection exclusively through the TL approach between 2006 and 2024—to our knowledge, the first dedicated series of the TL approach as the sole primary strategy in a homogeneous PPFM cohort. We characterized surgical, functional, and oncological outcomes and assessed whether anatomical extension patterns and tumor burden influenced extent of resection, FN outcomes, and postoperative morbidity. We hypothesized that favorable FN and functional outcomes would be achievable in selected patients, while extension patterns and tumor burden would differentially influence the burden of resection and complications.

## 2. Materials and Methods

### 2.1. Study Design and Patient Selection

This study was approved by the Institutional Review Board of the University of Wisconsin–Madison (IRB: 2024-1232), and the requirement for informed consent was waived given the retrospective design. We reviewed all patients who underwent resection of a posterior petrous surface meningioma via the TL approach at a single academic institution between 2006 and 2024, identified through a prospectively maintained institutional neurosurgical database. Inclusion required (1) histologically confirmed posterior petrous surface meningioma, (2) surgery performed exclusively via the TL approach, and (3) complete preoperative, operative, and postoperative data including neuroimaging and a minimum of one year of clinical follow-up. All clinical, radiological, and operative data were independently extracted by two reviewers, with discrepancies resolved by consensus with a senior author. Surgery was indicated for symptomatic lesions and/or documented radiographic progression on surveillance imaging.

### 2.2. Clinical and Radiological Variables

Preoperative variables collected included patient demographics (age, sex, body mass index), symptom duration, presenting neurological symptoms, and functional status. Tumor characteristics were assessed on preoperative contrast-enhanced magnetic resonance imaging (MRI) and included maximum tumor diameter and preoperative tumor volume. Tumor volume was calculated using the modified ellipsoid formula based on three orthogonal diameters obtained from contrast-enhanced MRI [23]. Petrosal surface involvement was classified as anterior, middle, and/or posterior zone—corresponding to the premeatal, meatal, and retromeatal categories of prior classifications [1,4,20,22]—based on the anatomical zone of dural attachment; zones were not mutually exclusive. IAC extension was recorded as present or absent. Preoperative hydrocephalus was defined as an Evans Index greater than 0.30 on axial MRI [24].

FN function was graded preoperatively and at discharge, 3 months, and 1 year postoperatively using the House–Brackmann (HB) grading scale [25], with grades I–II considered favorable and grades III–VI considered poor or moderate. Preoperative hearing status was classified as serviceable (pure-tone average ≤ 50 dB with speech discrimination score ≥ 50%) or non-serviceable according to the 1995 American Academy of Otolaryngology–Head and Neck Surgery (AAO-HNS) criteria [26]. Functional outcome was assessed using the modified Rankin Scale (mRS), with a score of 0–2 considered favorable [27], and the Glasgow Outcome Scale (GOS), with a score of 4–5 considered favorable [28].

### 2.3. Surgical and Outcome Definitions

All procedures were performed via the TL approach by a neurosurgeon and 2 neurotologists. Approach selection integrated audiological, radiologic, and anatomical factors: the TL corridor was chosen primarily for tumors with non-serviceable hearing or in whom hearing preservation was considered unlikely. In borderline cases with residual serviceable hearing, it was selected when audiologic predictors were unfavorable—absent auditory brainstem responses or markedly reduced speech discrimination—or when anatomical factors limited the likelihood of preservation, including large tumor volume, fundal extension, and complex IAC morphology. In these circumstances, complete and safe resection was prioritized over hearing preservation. Continuous intraoperative neurophysiological monitoring, including free-running and stimulated facial nerve electromyography, was used routinely throughout tumor dissection.

Extent of resection (EOR) was determined volumetrically on early postoperative MRI and defined as gross total resection (GTR, 100% resection), near-total resection (NTR, >95%), and subtotal resection (STR, 75–95%). Simpson grading [29] was assigned intraoperatively and recorded prospectively. Lumbar drain (LD) and/or external ventricular drain (EVD) placement was performed according to intraoperative and clinical judgment. Postoperative ventriculoperitoneal shunt placement was recorded separately.

Postoperative complications were documented prospectively and included cerebrospinal fluid (CSF) leak, defined as wound drainage, rhinorrhea, or otorrhea; postoperative hematoma; new or worsening cranial neuropathies; reoperation for any cause; and need for permanent CSF diversion. Postoperative MRI was evaluated for edema, gliosis, and infarction. Cerebral edema was defined as new T2/FLAIR hyperintensity on MRI obtained within 10 days of surgery. Gliosis was defined as persistent T2/FLAIR signal abnormality on follow-up MRI obtained at 3 months or later. Infarction was identified by restricted diffusion on diffusion-weighted imaging (DWI) or by chronic encephalomalacia on follow-up imaging. Tumor recurrence was defined as the appearance of new contrast-enhancing tumor following GTR on surveillance MRI. Tumor regrowth was defined as a measurable increase in residual tumor volume following NTR or STR. Adjuvant radiation therapy, hospital length of stay, and discharge disposition were recorded for all patients.

### 2.4. Statistical Analysis

All statistical analyses were performed using IBM SPSS Statistics (version 23.0; IBM Corp., Armonk, NY, USA). Continuous variables were summarized as medians with interquartile ranges (IQRs) after confirming non-normality using the Shapiro–Wilk test and visual inspection of boxplots, histograms, and Q–Q plots. Categorical variables were reported as counts and percentages.

Between-group comparisons for continuous variables were performed using the Mann–Whitney U test. Categorical variables were compared using Fisher’s exact test given the small sample size. Spearman’s rank correlation coefficient (ρ) was used to assess associations between continuous predictors and count or continuous outcomes, including complication count, operative duration, and hospital length of stay. The Mann–Whitney U test was additionally used to compare preoperative tumor volume between patients with and without specific postoperative complications. Because individual complications occurred too infrequently for separate analysis, the total number of complications per patient was additionally examined as a summary measure of overall perioperative burden; this composite aggregates events of differing severity and mechanism and was, therefore, treated as exploratory.

Univariate logistic regression was performed to identify factors associated with Simpson grade (grade 1–2 vs. 3–4), binary extent of resection (GTR + NTR vs. STR), and binary complication outcome (any complication vs. none). The results were expressed as odds ratios (ORs) with 95% confidence intervals (CIs). Complete or quasi-complete separation, which precludes reliable estimation of regression coefficients, was noted where applicable and reported as non-computable (NC). Multivariable regression analysis was not performed given the small cohort size and the limited number of outcome events, which precluded stable model estimation. FN outcome analysis at 1 year was limited to descriptive statistics in the preoperative HB I–II subgroup, as the absence of poor outcomes precluded regression modeling.

**Figure 1:**
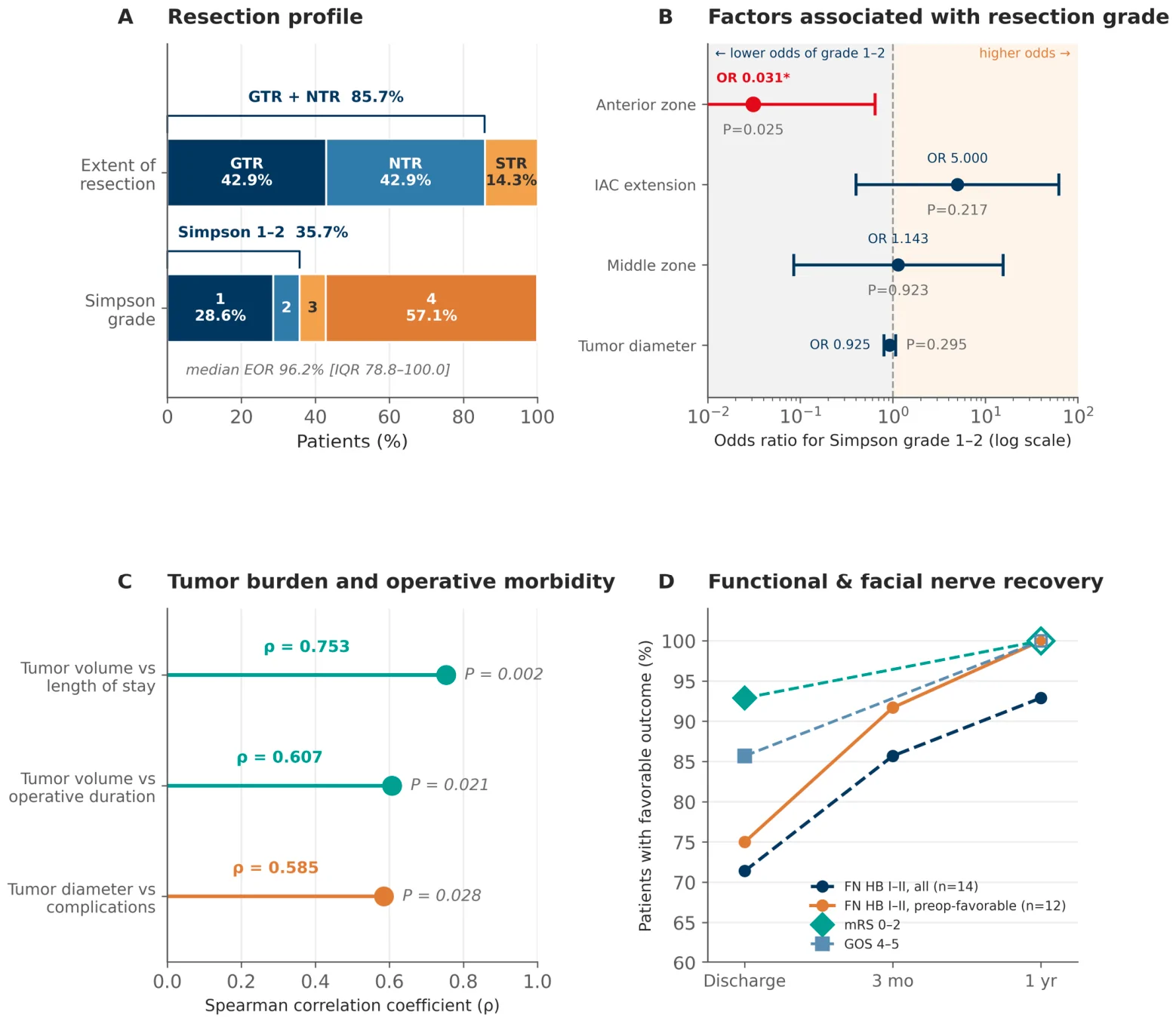
Surgical, functional, and oncological outcomes in 14 patients with posterior petrous face meningiomas treated via the translabyrinthine approach. (**A**) Distribution of extent of resection (top) and Simpson grade (bottom); brackets denote the combined GTR + NTR rate and Simpson grade 1–2 rate; median extent of resection was 96.2% (IQR 78.8–100.0). (**B**) Univariate logistic regression forest plot for factors associated with Simpson grade 1–2 resection, shown as odds ratios with 95% confidence intervals on a logarithmic scale; the dashed line denotes OR = 1, and the asterisk denotes statistical significance. (**C**) Spearman rank correlations between preoperative tumor burden and operative morbidity; each point represents a correlation coefficient (ρ) with its *p*-value. (**D**) Proportion of patients with favorable outcomes over time. House–Brackmann grade I–II facial nerve function is shown at discharge, 3 months, and 1 year for the entire cohort (*n* = 14) and for patients with preoperatively favorable facial nerve function (HB grade I–II, *n* = 12); modified Rankin Scale 0–2 and Glasgow Outcome Scale 4–5 are shown at discharge and 1 year. CI, confidence interval; GOS, Glasgow Outcome Scale; GTR, gross total resection; HB, House–Brackmann; IQR, interquartile range; mRS, modified Rankin Scale; NTR, near-total resection; OR, odds ratio; ρ, Spearman correlation coefficient; STR, subtotal resection.

Serial FN outcomes were compared across time points using the Wilcoxon signed-rank test, as were changes in mRS and GOS from admission to follow-up. A two-sided significance level of α = 0.05 was applied for all analyses. Given the limited number of patients and the exploratory and descriptive nature of this study, *p*-values were not corrected for multiple comparisons and should be interpreted as hypothesis-generating. Cohort surgical, functional, and oncological outcomes are summarized in Figure 1A–D.

## 3. Results

### 3.1. Patient Characteristics and Preoperative Status

A total of 14 patients were included. The median age was 61.0 years (IQR 52.5–68.0), and eight patients (57.1%) were female. The median duration of symptoms was 24.0 months (IQR 3.7–36.0), and recent clinical deterioration was observed in eight patients (57.1%). One patient (7.1%) had a recurrent tumor.

The most common presenting symptoms were hearing loss (71.4%), dizziness (64.3%), and gait imbalance (57.1%). Tinnitus was present in five patients (35.7%), and dysphagia, facial numbness, and headache were each present in four patients (28.6%). Less frequent symptoms included facial pain (21.4%), facial weakness and nausea/vomiting (each 14.3%), blurred vision (7.1%), and diplopia (7.1%). The median tumor diameter was 33.5 mm (IQR 27.5–42.0), and the median preoperative tumor volume was 8.5 cm^3^ (IQR 6.3–19.7). IAC extension was present in eight patients (57.1%), and preoperative hydrocephalus was identified in three patients (21.4%). Maximum tumor diameter ranged from 20 to 46 mm, and preoperative tumor volume ranged from 3.0 to 30.7 cm^3^. All patients were symptomatic at presentation; no lesion was detected incidentally. Favorable preoperative FN function (HB grade I–II) was observed in 12 patients (85.7%). Patient demographics and tumor characteristics are summarized in Table 1.

### 3.2. Hearing Status and Surgical Approach Selection

**Figure 2:**
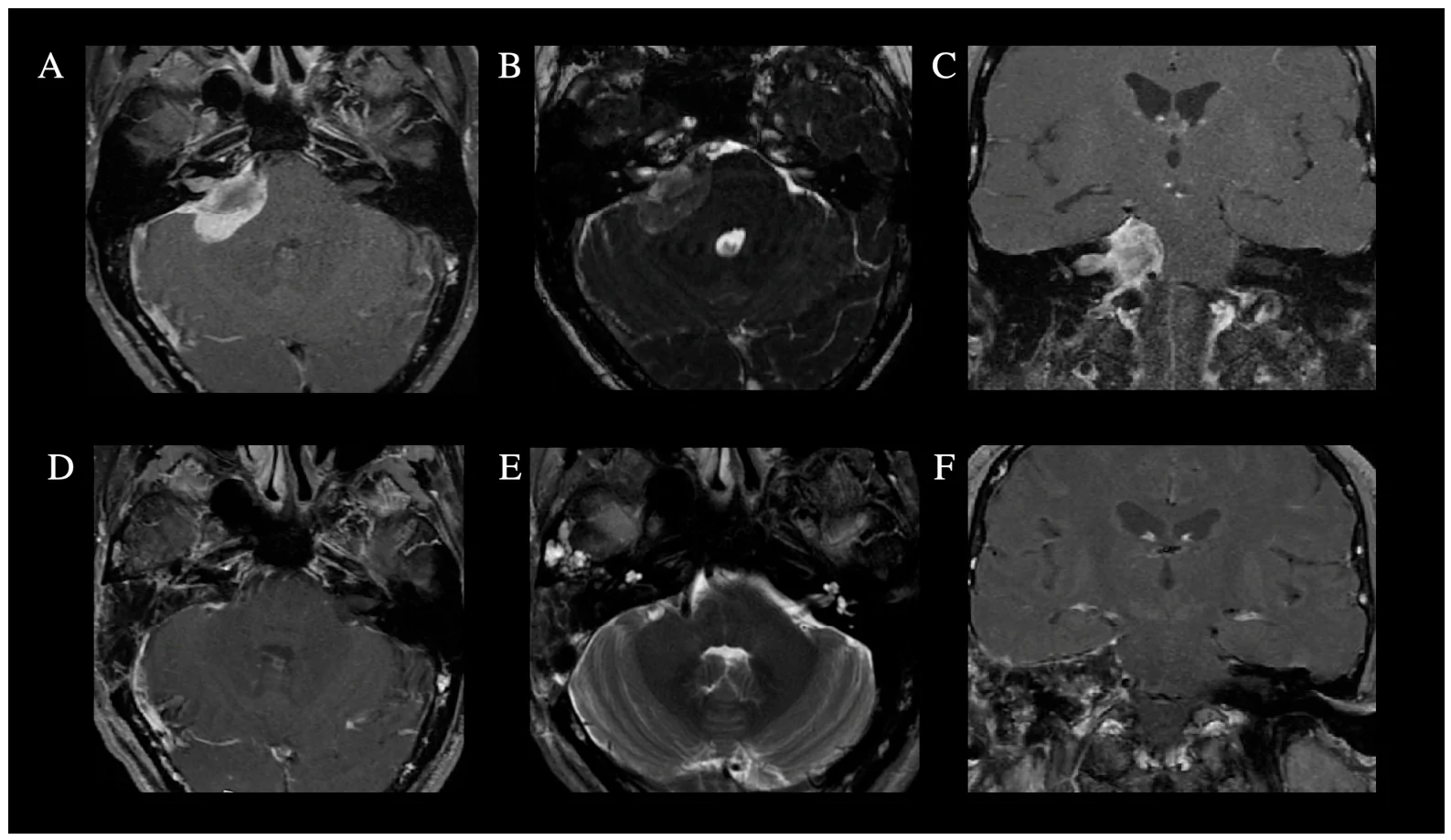
Illustrative Case 1—translabyrinthine gross total resection of a large, hemorrhagic right posterior petrous face meningioma presenting with multiple cranial neuropathies. A 62-year-old man presented acutely with sudden headache and right-sided cranial nerve V, VI, VII, VIII, and IX deficits, including complete facial palsy (House–Brackmann grade VI) and complete ipsilateral hearing loss. Imaging documented a hemorrhagic right cerebellopontine angle lesion with brainstem compression, histopathologically confirmed as a transitional meningioma involving all three petrosal zones with internal auditory canal (IAC) extension (maximum diameter 37 mm). Because hearing was already completely lost, a hearing-ablative corridor carried no additional auditory cost; the translabyrinthine approach was, therefore, selected over the retrosigmoid route to provide direct lateral-to-medial access to this large, multi-zone lesion, early facial nerve identification at the meatal fundus, and exposure of the posterior petrous dural attachment for early devascularization without cerebellar retraction. Preoperative images (**A**–**C**): axial contrast-enhanced T1-weighted (**A**), axial T2-FLAIR (**B**), and coronal contrast-enhanced T1-weighted (**C**) images show the enhancing tumor with IAC involvement and anterior petrosal extension. Postoperative images (**D**–**F**): axial fat-suppressed contrast-enhanced T1-weighted (**D**), axial fat-suppressed T2-weighted (**E**), and coronal fat-suppressed contrast-enhanced T1-weighted (**F**) images show the translabyrinthine cavity obliterated by an abdominal fat graft, with no residual enhancing tumor consistent with gross total resection. Preoperative hydrocephalus was managed with an external ventricular drain followed by postoperative ventriculoperitoneal shunting, and transient postoperative swallowing difficulty resolved with therapy. Facial nerve function improved progressively from House–Brackmann grade VI preoperatively to grade III at 1 year, with a favorable global outcome (modified Rankin Scale 1, Glasgow Outcome Scale 5).

**Figure 3:**
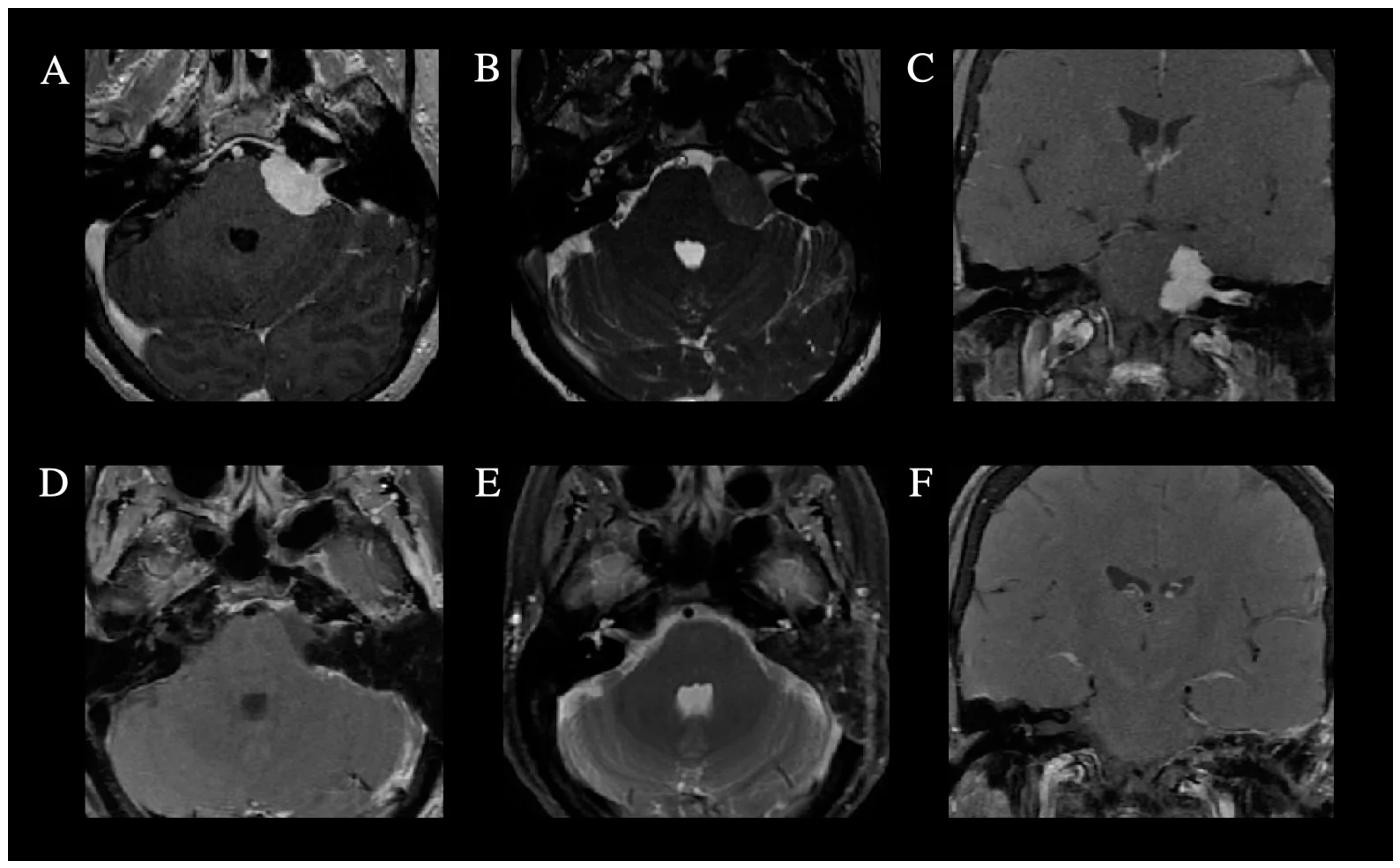
Illustrative Case 2—translabyrinthine gross total (Simpson grade 1) resection of a left posterior petrous face meningioma with preserved facial nerve function. A 60-year-old woman presented with mild asymmetric left-sided sensorineural hearing loss. Although hearing remained measurable, poor left-sided speech discrimination and an abnormal auditory brainstem response (prolonged wave I–III interval with absence of waves III and V) indicated retrocochlear dysfunction, while normal tympanometry excluded a conductive component. Contrast-enhanced MRI demonstrated a sharply marginated, homogeneously enhancing left cerebellopontine angle lesion without hemorrhage or restricted diffusion, extending into the porus acusticus and central internal auditory canal (IAC) with mild mass effect on the adjacent brainstem; the lesion was histopathologically confirmed as a secretory meningioma involving the anterior, middle, and posterior petrosal zones (maximum diameter 30 mm). Given these unfavorable audiologic predictors together with IAC involvement, meaningful hearing preservation was considered unlikely; priority was, therefore, placed on maximal safe resection and facial nerve preservation, and after counseling, the translabyrinthine approach was selected for its direct lateral access and early facial nerve identification at the meatal fundus. Preoperative images (**A**–**C**): axial contrast-enhanced T1-weighted (**A**), axial T2-FLAIR (**B**), and coronal contrast-enhanced T1-weighted (**C**) images show the enhancing tumor at the IAC. Postoperative images (**D**–**F**): axial fat-suppressed contrast-enhanced T1-weighted (**D**), axial fat-suppressed T2-weighted (**E**), and coronal fat-suppressed contrast-enhanced T1-weighted (**F**) images show the translabyrinthine cavity obliterated by an abdominal fat graft, with no residual enhancing tumor consistent with gross total resection. Resection was Simpson grade 1, facial nerve function remained House–Brackmann grade I throughout, and the postoperative course was uncomplicated.

**Figure 4:**
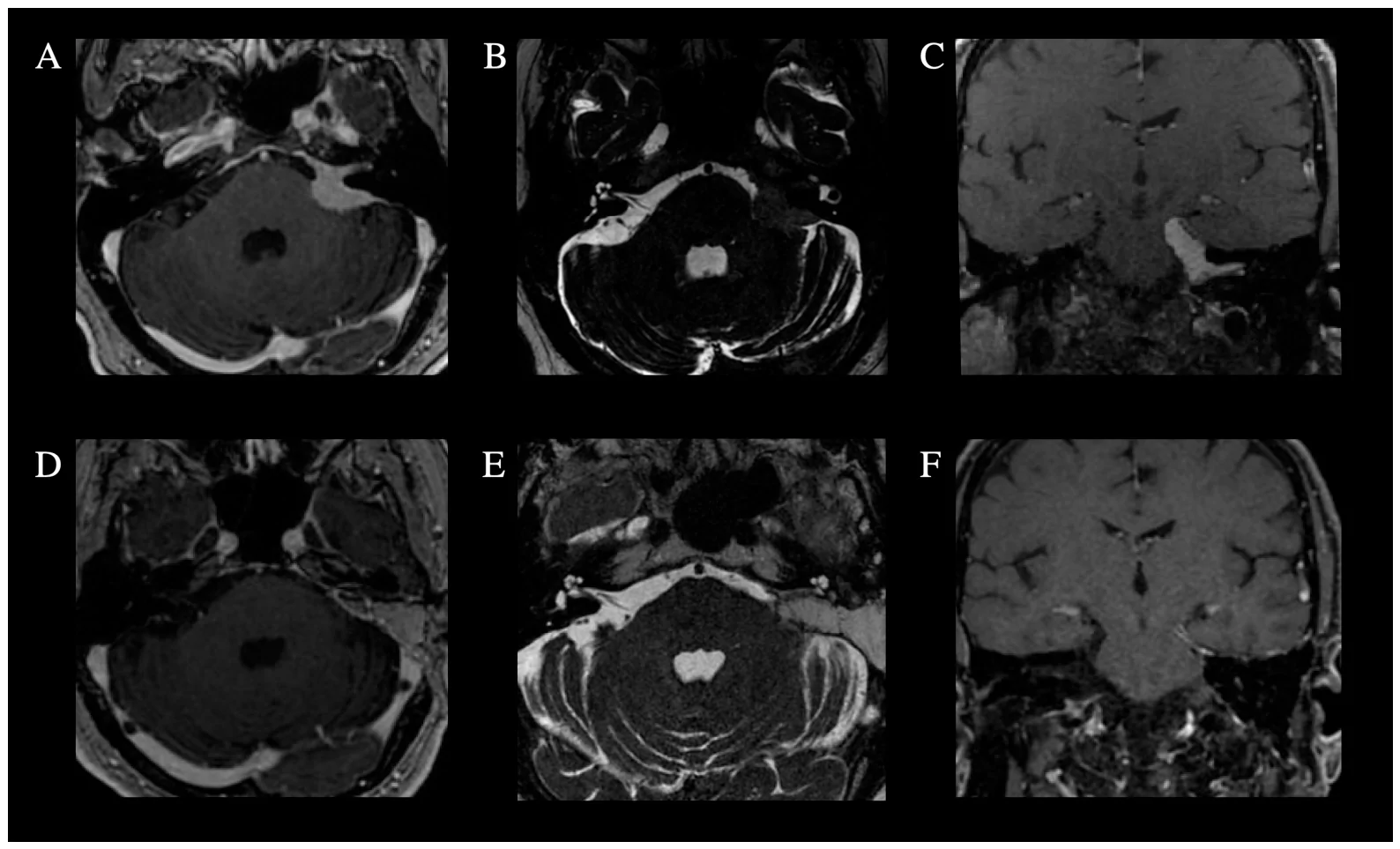
Illustrative Case 3—translabyrinthine gross total (Simpson grade 1) resection of a left posterior petrous face atypical (WHO grade II) meningioma with preserved facial nerve function. A 56-year-old man presented with nine months of progressive left-sided hearing loss, tinnitus, aural fullness, and imbalance. Audiometry demonstrated moderate-to-severe left sensorineural hearing loss, with word recognition untestable owing to its severity, indicating non-serviceable hearing. With no useful hearing to preserve and a tumor extending well into the internal auditory canal (IAC), the translabyrinthine approach was selected for direct lateral access and early facial nerve identification at the meatal fundus. Contrast-enhanced MRI demonstrated a dural-based left cerebellopontine angle meningioma (maximum diameter 37 mm) predominantly involving the posterior and middle petrosal zones, with significant IAC extension and encasement of the left seventh and eighth (facial and vestibulocochlear) cranial nerves. Preoperative images (**A**–**C**): axial contrast-enhanced T1-weighted (**A**), axial T2-FLAIR (**B**), and coronal contrast-enhanced T1-weighted (**C**) images show the enhancing tumor at the IAC. Postoperative images (**D**–**F**): axial contrast-enhanced T1-weighted (**D**), axial T2-weighted (**E**), and coronal fat-suppressed contrast-enhanced T1-weighted (**F**) images show the translabyrinthine cavity obliterated by an abdominal fat graft, with no residual enhancing tumor consistent with gross total resection. Histopathology confirmed an atypical (WHO grade II) meningioma, and adjuvant radiation therapy was administered. Resection was Simpson grade 1, facial nerve function remained House–Brackmann grade I throughout, and the postoperative course was uncomplicated.

Of the 14 patients, 10 (71.4%) had non-serviceable or absent hearing preoperatively (AAO-HNS Class C–D), while four (28.6%) had residual hearing (AAO-HNS Class B). In the latter, the TL approach was selected for borderline hearing when audiologic predictors were poor—including abnormal electrophysiology (retrocochlear dysfunction on auditory brainstem response)—or when anatomical factors limited hearing preservation, such as large tumor volume, fundal extension, and complex IAC morphology, rather than with an intent to preserve hearing (Figure 2, Figure 3 and Figure 4).

### 3.3. Surgical Parameters and Extent of Resection

All patients underwent a TL approach. The median operative time was 576.5 min (IQR 501–713). GTR was achieved in six patients (42.9%), NTR in six patients (42.9%), and STR in two patients (14.3%), with a median EOR of 96.2% (IQR 78.8–100.0). According to Simpson grading, five patients (35.7%) had grade 1–2 resection, whereas nine patients (64.3%) had grade 3–4 resection (Table 2) (Figure 1A). Histopathology confirmed WHO grade I meningioma in 11 patients (78.6%) and WHO grade II in three patients (21.4%); no grade III tumors were identified (Table 1).

### 3.4. Cerebrospinal Fluid Diversion and Hydrocephalus Management

Preoperative hydrocephalus was present in three patients (21.4%). Among these, one patient underwent preoperative EVD placement followed by postoperative ventriculoperitoneal shunt insertion. One patient was managed with intraoperative LD placement to facilitate brain relaxation and did not require postoperative CSF diversion, whereas the remaining patient required postoperative shunt placement without prior CSF diversion.

Among patients without preoperative hydrocephalus, intraoperative LD placement was performed in two patients to facilitate CSF drainage and surgical exposure. Both patients subsequently required postoperative ventriculoperitoneal shunt placement. In one, shunt placement followed a postoperative CSF leak that developed despite the lumbar drain.

### 3.5. Functional Outcomes

At discharge, 13 patients (92.9%) had favorable functional outcomes (mRS score 0–2), and all patients (100%) sustained favorable outcomes at 1-year follow-up. GOS score 4–5 was achieved in 12 patients (85.7%) at discharge and in all 14 patients (100%) at 1-year follow-up. FN function demonstrated progressive improvement over time, with HB grade I–II observed in 10 patients (71.4%) at discharge, 12 patients (85.7%) at 3 months, and 13 patients (92.9%) at 1 year (Figure 1D).

Among patients with preoperative HB I–II (*n* = 12), nine patients (75.0%) retained HB I–II at discharge, 11 patients (91.7%) at 3 months, and all 12 patients (100%) at 1 year. Cranial nerve VI palsy was present preoperatively in one patient (7.1%) and postoperatively in three patients (21.4%), including one preexisting case. The two new postoperative deficits resolved by 1-year follow-up, and both occurred in patients with anterior petrosal zone involvement.

Dysphagia was present preoperatively in four patients (28.6%). In the early postoperative period, dysphagia improved in one patient and persisted in three patients who required swallowing therapy. At 1-year follow-up, dysphagia had resolved in all affected patients.

No variables were significantly associated with FN outcome at 1 year (all *p* > 0.05). In patients with preoperative HB I–II, all patients retained HB I–II at 1 year (100%), precluding further regression analysis.

### 3.6. Factors Associated with Resection and Simpson Grade

No significant associations were identified between extent of resection and tumor location, IAC extension, hydrocephalus, or recurrent status (all *p* > 0.05). Similarly, tumor diameter and preoperative tumor volume were not significantly associated with extent of resection (*p* = 0.791 and *p* = 0.923, respectively).

In contrast, anterior petrosal zone involvement was significantly associated with Simpson grade (*p* = 0.023), with Simpson grade 3–4 more frequently observed in tumors with anterior zone involvement. Univariate logistic regression identified anterior petrosal zone involvement as the only variable significantly associated with a lower likelihood of Simpson grade 1–2 resection (OR 0.031, 95% CI 0.002–0.641, *p* = 0.025) (Table 3) (Figure 1B). Notably, anterior zone involvement was not associated with volumetric extent of resection, indicating that it influenced completeness of dural resection rather than the volume of tumor removed. No other variables were significantly associated with Simpson grade (all *p* > 0.05).

### 3.7. Postoperative Complications

Postoperative complications occurred in eight patients (57.1%); several patients experienced more than one complication. These comprised ventriculoperitoneal shunt placement in four patients (28.6%), postoperative dysphagia requiring swallowing therapy in three patients (21.4%), new abducens (cranial nerve VI) palsy in two patients (14.3%), and CSF leak in two patients (14.3%). One patient underwent reoperation for an isolated wound complication after gross total resection, in the absence of CSF leak or CSF diversion. No postoperative hematomas or systemic complications were observed (Table 4). Infarction was identified on postoperative imaging in one patient (7.1%) without a corresponding neurological deficit.

No variables were significantly associated with any complication as a binary outcome (all *p* > 0.05). Larger tumor size demonstrated a non-significant trend toward increased complication rates (*p* = 0.081), whereas IAC extension showed a non-significant trend toward fewer complications (OR 0.120, *p* = 0.107). Preoperative tumor volume was significantly associated with postoperative CSF leak (Mann–Whitney U = 0.000, *p* = 0.022) (Table 5) (Figure 1C).

### 3.8. Exploratory Analyses of Postoperative Complications

No variable was significantly associated with the occurrence of any complication as a binary outcome (all *p* > 0.05); larger tumor size showed a non-significant trend (*p* = 0.081). In exploratory analyses using the total number of complications per patient as a summary measure, tumor diameter demonstrated a positive correlation with complication count (Spearman ρ = 0.585, *p* = 0.028), and preoperative hydrocephalus was associated with a higher complication count (mean rank 11.83 vs. 6.32, *p* = 0.030).

Preoperative tumor volume demonstrated significant correlations with operative duration (ρ = 0.607, *p* = 0.021) and length of hospital stay (ρ = 0.753, *p* = 0.002) and showed a non-significant correlation with complication count (ρ = 0.453, *p* = 0.104) (Table 5).

### 3.9. Tumor Control

Over a median follow-up of 65.5 months (IQR 23.7–112.7), tumor recurrence occurred in one patient (7.1%) following gross total resection, and tumor regrowth was observed in three patients (21.4%) in the near-total or subtotal resection subgroup. Adjuvant radiation therapy was administered to five patients (35.7%) overall: for postoperative recurrence (one patient), for regrowth of residual tumor (two patients), for subtotal resection with residual disease (one patient), and for atypical (WHO grade II) histology (one patient); the remaining regrowth was managed with observation and serial imaging. Tumor control in this cohort, therefore, reflects multimodal management rather than surgical resection alone. No clinical or radiological variable was significantly associated with recurrence or regrowth (all *p* > 0.05).

## 4. Discussion

In this single-institution series of 14 consecutive PPFMs treated exclusively through the TL approach, we characterized surgical, functional, and oncological outcomes within the first dedicated single-corridor cohort reported to date. Three principal observations emerged. First, TL resection was associated with favorable functional outcomes, a high rate of GTR/NTR, and no perioperative mortality; all patients with preoperative HB I–II facial nerve function retained favorable function at 1 year. Second, anterior petrosal zone involvement was not associated with volumetric extent of resection but was associated with a lower likelihood of Simpson grade 1–2 resection, suggesting greater difficulty in addressing the anterior dural attachment and involved bone through a lateral corridor. Third, measures of tumor burden, including tumor diameter, volume, and preoperative hydrocephalus, were associated with postoperative complication burden and perioperative outcomes. During a median follow-up of 65.5 months, one recurrence after GTR and three cases of residual tumor regrowth were observed. Collectively, these findings suggest that anatomical extension and tumor burden were associated with different aspects of surgical outcome in TL-treated PPFMs—Simpson grade and postoperative morbidity, respectively.

### 4.1. Translabyrinthine Approach in Posterior Petrous Face Meningiomas

Although the TL approach was initially developed and refined for vestibular schwannoma surgery, its application has progressively expanded to other lesions of the CPA and petrous region, including meningiomas. First attempted in the early twentieth century, the corridor was largely abandoned for several decades before being reestablished as a safe route for CPA tumor surgery by the mid-1960s [30,31,32]. Early reports of petrous meningioma surgery emphasized that corridor selection should be guided by tumor location and extension, positioning the TL route as one of several complementary approaches to the petrous region rather than a universally applicable strategy [33]. Subsequent refinements, including extended TL and combined subtemporal–TL approaches, broadened its application to more complex lesions extending toward the petrous apex and adjacent skull base compartments [34].

More recently, increasing recognition of the heterogeneity within petrous region meningiomas has emphasized the importance of anatomy-specific classification and surgical decision-making. Among these distinctions, the separation of PPFMs from petroclival meningiomas has received particular attention because of its prognostic implications, with the trigeminal nerve marking the boundary between the two [8,35,36]. PPFMs should, however, be regarded more broadly as a distinct subgroup within the heterogeneous spectrum of petrous region meningiomas, characterized by specific patterns of neurovascular displacement [37], clinical presentation [38,39], and operative considerations. Despite these advances, dedicated PPFM cohorts have remained predominantly retrosigmoid based, whereas experience with the TL corridor has largely been embedded within anatomically heterogeneous skull base series that combined PPFMs with petroclival, petrous apex, petrotentorial, jugular foramen, and other skull base meningiomas. The present study addresses the intersection of these two underexplored literature streams by evaluating the TL approach exclusively within a homogeneous cohort of PPFMs, providing a defined denominator against which corridor-specific outcomes can be interpreted.

### 4.2. Surgical Corridor Selection and Comparative Framework

To situate the TL corridor within the broader management of petrous region meningiomas, we synthesized established anatomical classification systems and published surgical approach principles into an anatomy-based comparative framework (Table 6) [1,4,20,22,35]. Within this framework, PPFMs are subdivided according to their relationship to the IAC into premeatal, meatal, and retromeatal variants, each carrying distinct implications for corridor selection [1,4,15,20]. Retromeatal lesions, situated posterior to the IAC with the facial–vestibulocochlear complex displaced anteriorly, are well suited to the retrosigmoid approach and permit hearing preservation when the fundus is spared [1,5,15]. Meatal lesions, centered on the IAC with frequent fundal involvement, place the FN at greatest risk and favor the TL corridor, which provides direct lateral-to-medial access and early FN identification at the meatal fundus before tumor dissection [4,9,10]. Premeatal lesions, extending anterior to the IAC toward the petrous apex, may mimic petroclival extension and have been associated with less favorable functional outcomes [20]. Depending on hearing status and the predominant direction of growth, these lesions may be managed through the TL corridor or may require extension toward anterior approaches [4,11].

The framework further delineates PPFMs from anatomically adjacent entities for which the TL corridor alone is insufficient. Petroclival meningiomas, arising medial to the trigeminal nerve and displacing it posterolaterally, extend toward the clivus and brainstem and often require combined transpetrosal or presigmoid corridors, with the TL approach contributing primarily as the posterior limb of a combined exposure [8,36,40]. Anterior petrous and petrous apex lesions typically require an anterior trajectory through a Kawase or extended middle fossa corridor, as this deep-seated territory lies beyond the reach of a purely lateral approach [11,12,13]. Petrotentorial, petro-occipital, and intrapetrous lesions similarly require supratentorial, far-lateral, or tailored combined approaches according to their dural epicenter and pattern of bony involvement [7]. This delineation reinforces that PPFMs should be managed according to their specific dural attachment and pattern of extension rather than a uniform algorithm [4,6]. When appropriately selected on anatomical and audiological grounds, the TL approach can achieve favorable functional and oncological outcomes, without implying corridor superiority.

### 4.3. Anatomical Heterogeneity and Extent of Resection

The anatomical heterogeneity within our cohort is consistent with prior observations that PPFMs are not a uniform entity [1,4,6]. Only four of fourteen patients (28.6%) had tumors confined to a single petrosal zone, whereas the remaining ten (71.4%) demonstrated multi-zone involvement (Table 1). Several classification systems—including those proposed by Desgeorges et al. [22], Schaller et al. [20], Sanna et al. [4], and Donofrio et al. [1]—have addressed this heterogeneity but assign each tumor to a single dominant or combined subtype, whether an overlapping category such as anterior–middle–posterior or a broad-based designation. Prior petrous face series applying the Desgeorges scheme have likewise grouped multi-zone tumors into a single combined category for analysis [6]. Because such grouping collapses overlapping tumors and may obscure the independent contribution of each anatomical component, we instead modeled involvement of the anterior, middle, and posterior petrosal zones as three independent binary variables, thereby permitting separate evaluation of each anatomical component within this cohort.

The association between anterior petrosal zone involvement and Simpson grade 3–4 resection (Table 3) has a plausible anatomical basis within the TL corridor. The anterior petrosal zone abuts the petrous apex and the petroclival junction, where the dural plane and underlying hyperostotic bone lie at the extreme medial reach of a lateral-to-medial trajectory. Although the TL corridor provided excellent volumetric exposure of the posterior fossa component—reflected in a combined GTR and NTR rate of 85.7% (Table 2)—complete extirpation of the anterior dural attachment and adjacent hyperostotic bone, often necessary for Simpson grade 1–2 resection, may be limited by working depth and angle of attack. This distinction explains why anterior zone involvement affected Simpson grade without reducing volumetric extent of resection. Despite the high rate of GTR and NTR, Simpson grade 1–2 resection was observed less frequently (35.7%) and predominantly in patients without anterior petrosal zone involvement (Figure 1B). This surgical constraint aligns with prior data indicating that lesions more medially located relative to the trigeminal nerve are associated with lower rates of complete resection [8,35]. Within the TL corridor, the previously described transapical extension [4] may partially overcome this limitation, although at the cost of additional drilling near the petrous internal carotid artery; alternatively, an anterior transpetrosal (Kawase) corridor or a combined approach can provide a more direct trajectory to the anterior petrosal dural attachment [11,12,13]. Notably, no clinical or radiological variable in our series was significantly associated with long-term recurrence or regrowth (all *p* > 0.05). Because five patients (35.7%) received adjuvant radiation therapy, the observed recurrence and regrowth rates reflect multimodal management rather than surgical resection alone. Within these limits, the findings suggest that high volumetric clearance, combined with adjuvant radiation where indicated, may provide acceptable intermediate-term tumor control despite residual microscopic dural disease in selected cases, although longer follow-up is warranted.

### 4.4. Postoperative Complications and Tumor Burden

In contrast to extent of resection, which was associated principally with anatomical extension, postoperative complication burden was associated principally with measures of tumor burden (Table 4 and Table 5). Maximum tumor diameter correlated with the number of postoperative complications (ρ = 0.585, *p* = 0.028), and preoperative tumor volume correlated with operative duration (ρ = 0.607, *p* = 0.021) and length of hospital stay (ρ = 0.753, *p* = 0.002) (Figure 1C). Larger tumors more frequently distort the regional neurovascular anatomy, compress the brainstem, and disrupt the arachnoid planes that normally facilitate safe dissection, thereby prolonging operative time and recovery [2,8]. The same volumetric burden was specifically associated with CSF leak, likely reflecting the more extensive dural opening and petrous bone work required for larger lesions, with greater exposure of mastoid air cells [10]. This association should be interpreted with caution, however, as CSF leak occurred in only two of fourteen patients, and a single additional event would substantially alter the estimate. Preoperative hydrocephalus was likewise associated with a higher cumulative complication burden, which may reflect altered CSF dynamics related to mass effect together with the added morbidity of CSF diversion procedures.

These associations are broadly supported by the wider literature, although the specific predictors have varied across series. In a meta-analysis stratifying outcomes by dural origin, larger posterior petrous meningiomas (≥3.5 cm) showed a significantly higher prevalence of cranial nerve deficit, identifying tumor size as a determinant of morbidity within this subgroup [35]. Conversely, a retrosigmoid posterior petrous meningioma series did not identify maximum tumor diameter as a predictor of postoperative complications [6], underscoring that the influence of tumor burden may be modulated by surgical corridor and cohort composition. Our overall CSF leak rate (14.3%) exceeded the rates of approximately 6.9–9.8% reported in retrosigmoid posterior petrous meningioma series [5,21] as well as the overall rate of 6.9% reported in a mixed transpetrosal cohort that included retrosigmoid, TL, and other approaches [10]. This difference may, at least in part, reflect the obligatory mastoid and petrous bone drilling intrinsic to the TL approach [10], compounded by the small cohort size and the predominance of large tumors. Reassuringly, no perioperative deaths occurred, and the majority of complications, including all cases of lower cranial nerve dysfunction, resolved during follow-up, consistent with the low mortality and largely transient morbidity reported across contemporary PPFM series [1,4].

Notably, neither IAC extension nor anatomical zone involvement was associated with complication burden, reinforcing that anatomical extension and tumor burden act through distinct pathways—the former constraining resection, the latter driving morbidity. Accordingly, measures of tumor burden may inform perioperative planning, as larger tumors were associated with longer operative duration, prolonged hospitalization, and an increased likelihood of CSF leak, whereas preoperative hydrocephalus warrants careful consideration of CSF diversion strategies.

### 4.5. Facial and Cranial Nerve Outcomes

FN preservation represented the most favorable functional outcome in this cohort. All 12 patients with preoperative House–Brackmann (HB) grade I–II function continued to exhibit HB grade I–II function at 1 year, and 92.9% of the cohort overall demonstrated favorable FN function at final follow-up. These outcomes compare favorably with the published literature, although direct comparisons remain challenging because of differences in tumor location, outcome definitions, and surgical corridors. HB grade I–II function has been reported in 88.9% of patients undergoing retrosigmoid resection of cerebellopontine angle meningiomas [15] and in 81% of cases in a predominantly retrosigmoid posterior petrous series of 82 patients [17]. Among cohorts incorporating TL-based approaches, a mixed transpetrosal series combining retrolabyrinthine, TL, and other corridors reported normal-to-near-normal function in 73% of patients with posterior petrous meningiomas [10], a temporal bone meningioma series reported satisfactory (HB grade I–II) function in 64% of its TL subgroup [9], and anatomically heterogeneous cohorts of petroclival and posterior petrous meningiomas have reported favorable TL-specific FN preservation in approximately 58% to 83% of patients [35]. Although these comparisons should be interpreted cautiously, the relative scarcity of TL-specific data in homogeneous PPFM cohorts highlights the significance of the favorable outcomes observed in the present study.

The favorable FN outcome in our cohort is anatomically grounded in the TL corridor itself. Because the approach proceeds in a lateral-to-medial direction, the FN is identified early at the meatal fundus using stable bony landmarks before tumor dissection begins, rather than after tumor debulking and mobilization as in the retrosigmoid approach [3]. Early nerve identification, meticulous preservation of the arachnoid plane, and avoidance of excessive traction during dissection facilitate continuous protection of the nerve throughout tumor removal. The anatomy of PPFMs may further reinforce this advantage: because these tumors characteristically displace the facial–vestibulocochlear complex anteriorly or anteromedially, the FN is typically displaced away from the principal line of dissection, thereby reducing the likelihood of direct nerve manipulation during resection [3].

The same anatomical features that favor facial nerve preservation also govern vascular safety within this corridor. This distinction follows from the site of dural origin: petroclival meningiomas arise medial to the trigeminal nerve and are separated from the brainstem by a single prepontine arachnoid layer, so that adhesion to the brainstem and its perforators is common; PPFMs arise lateral to the nerve and retain the multilayered arachnoid contributed by the prepontine, ambient, and crural cisterns [8]. This preserved plane keeps the anterior inferior cerebellar artery and its branches, together with the lower cranial nerves, on the far side of the dissection and facilitates intra-arachnoidal separation. Exposure was obtained by bone removal rather than by brain retraction: no fixed cerebellar retractor was used, since retraction of the cerebellum against the petrous surface is a principal mechanism of vascular and venous injury in this region. Early devascularization at the posterior petrous dural attachment permitted internal debulking before capsular dissection, reducing traction on the peritumoral vasculature and allowing the capsule to be drawn toward the surgeon rather than toward the brainstem. The AICA and its branches were identified and preserved along the inferior and anterior tumor poles through subarachnoid-plane dissection with continuous intraoperative neuromonitoring, so that vascular structures were encountered after volume reduction and separated by sharp dissection rather than under tension. Where tumor abutted an arterial loop or perforator, the vessel was released by sharp circumferential dissection under high magnification; traction was not applied in order to achieve complete separation, and vessel patency was confirmed before closure.

Other cranial nerve deficits were less common and were generally transient. A new abducens nerve deficit occurred in two patients (14.3%), in addition to one preexisting deficit; both new deficits resolved by 1-year follow-up. This observation is anatomically plausible, given the close relationship of the abducens nerve and Dorello canal to the petrous apex and petroclival junction, regions reached only at the extreme medial extent of the lateral-to-medial trajectory and frequently approached when tumors extend anteriorly along the petrous surface, as both affected tumors did in our cohort [8]. These transient deficits may, therefore, reflect the vulnerability of the abducens nerve during dissection of anteriorly extending tumors rather than direct injury within the principal surgical corridor, consistent with the lower abducens morbidity reported for PPFMs relative to petroclival meningiomas [8].

Postoperative dysphagia requiring swallowing therapy occurred in three patients (21.4%), all of whom had preoperative dysphagia and all of whom recovered within 1 year; no patient developed new postoperative dysphagia. Because PPFMs characteristically spare the jugular foramen, these findings do not represent direct neural invasion. Instead, in larger tumors, the inferior tumor pole may displace the lower cranial nerve complex caudally and lie in close relation to the anterior inferior cerebellar artery and its branches; the transient dysfunction observed likely reflects traction or manipulation of this neurovascular complex during separation of the inferior capsule from the surrounding cisterns rather than nerve transection, consistent with the temporary lower cranial nerve dysfunction reported in retrosigmoid PPFM series [3,5,10]. Preoperative dysphagia was observed in patients with numerically larger tumors (median volume 13.2 vs. 8.5 cm^3^; median maximum diameter 39.5 vs. 31.0 mm), although this difference was not statistically significant in this small cohort. This pattern is consistent with caudal displacement of the lower cranial nerve complex by the inferior tumor pole rather than with posterior petrosal zone involvement or direct neural invasion.

Preoperative trigeminal symptoms were present in a minority of patients and, where present, likely reflected anterior extension toward Meckel cave. No new postoperative trigeminal deficits occurred, and all preoperative symptoms resolved during follow-up. The lateral-to-medial trajectory of the TL corridor may contribute to this favorable outcome by allowing the trigeminal nerve to remain outside the primary line of dissection. Collectively, these findings suggest that, in appropriately selected patients, TL resection of PPFMs can achieve favorable FN preservation with acceptable rates of predominantly transient non-facial cranial nerve morbidity.

### 4.6. Residual Hearing and Corridor Selection

The decision to employ a hearing-ablative corridor warrants justification because the TL approach necessarily sacrifices residual labyrinthine function. In 10 of 14 patients (71.4%) with non-serviceable or absent hearing, the TL approach carried no additional auditory cost while providing direct exposure of the posterior petrous surface and IAC. The remaining four patients (28.6%) retained residual hearing; however, residual hearing does not necessarily imply functional hearing or a realistic likelihood of preservation, and in these patients unfavorable audiological and tumor-related features rendered meaningful preservation unlikely, so maximal safe resection was prioritized.

This selective strategy is consistent with the broader literature on posterior petrous meningioma surgery. Although the retrosigmoid approach offers the opportunity for hearing preservation and remains favored for retromeatal tumors associated with serviceable hearing [15,20], this possibility becomes increasingly limited in premeatal and anteriorly extending lesions. Dedicated posterior petrous face series have demonstrated that such tumors frequently present with non-serviceable hearing and are often managed through TL-based corridors, with hearing-preserving surgery reserved for a minority of appropriately selected patients [4,19]. Indeed, total tumor removal has been prioritized over hearing preservation in the largest dedicated PPFM series employing TL-based approaches [4]. Furthermore, preoperative tumor size alone has not consistently predicted postoperative hearing outcomes [15], suggesting that corridor selection should integrate audiological status, tumor anatomy, and the realistic likelihood of preserving useful hearing rather than relying on any single variable in isolation. Collectively, these observations indicate that residual hearing, by itself, should not preclude a TL approach when audiological and anatomical factors argue against a meaningful chance of hearing preservation and when wider exposure may facilitate safer resection. This principle is reflected in the present series, in which the TL approach was selected on the basis of non-serviceable hearing and anatomical features—including internal auditory canal involvement and multi-zone extension—with gross total resection achieved while preserving or improving facial nerve function in the representative cases (Figure 2, Figure 3 and Figure 4).

### 4.7. Clinical Implications

These findings carry several practical implications. Rather than supporting a uniform strategy, they suggest that operative planning should integrate anatomical extension, tumor burden, audiological status, and the functional consequences of the selected corridor. Because anterior petrosal zone involvement was the only significant correlate of Simpson grade 3–4 resection, recognizing anterior extension may calibrate expectations for a lateral corridor and prompt consideration of adjunctive or alternative approaches when Simpson grade 1–2 resection is a priority. Conversely, because postoperative morbidity tracked tumor burden rather than extension, larger tumor diameter and volume—and preoperative hydrocephalus—may inform perioperative counseling and CSF diversion planning. Finally, the favorable FN outcomes support the TL approach in appropriately selected patients, particularly when hearing is non-serviceable or unlikely to be preserved; residual hearing alone should not dictate corridor selection. Accordingly, we do not advocate a uniform preference for either corridor: for retromeatal tumors with serviceable, preservable hearing, the retrosigmoid approach remains preferred, whereas the TL corridor is preferred when hearing is non-serviceable or unlikely to be preserved and when IAC or fundal involvement favors direct lateral access. Overall, these observations support individualized corridor selection tailored to each patient’s anatomical, functional, and audiological characteristics.

### 4.8. Study Limitations

This study has several limitations that warrant consideration. First, the cohort comprised 14 patients treated at a single institution using the same surgical corridor by a neurosurgeon and two neurotologists. Although this consistency minimizes variability related to surgeon preference and technique, it inevitably limits statistical power and the generalizability of the findings. The small sample size and the relatively low frequency of certain events—including recurrence, regrowth, and individual cranial nerve deficits—constrained the statistical analyses; several models did not converge or yielded wide confidence intervals, and the resulting estimates should, therefore, be interpreted with appropriate caution. Second, the retrospective design carries the attendant risks of incomplete documentation and selection bias, despite the use of a prospectively maintained database and independent dual data extraction.

Third, and most importantly, the TL approach was applied selectively to a population defined in large part by non-serviceable hearing and specific anatomical features. This confounding by indication precludes direct comparison of surgical corridors and means that our findings cannot establish the superiority of the TL approach over the retrosigmoid or other alternatives; rather, they characterize the outcomes achievable when the TL corridor is selected on appropriate anatomical and audiological grounds. A randomized or matched comparison across corridors would be required to address relative efficacy, although such studies are unlikely to be feasible given the rarity of these tumors and the anatomically driven nature of approach selection. Fourth, the Simpson grading system was derived from a predominantly supratentorial cohort, in which wide excision of the dural attachment is often achievable. On the posterior petrous surface, this is rarely feasible: the attachment lies over the petrous bone in immediate relation to the sigmoid and superior petrosal sinuses, the jugular bulb, and the IAC, so that grade 1 resection is anatomically constrained and the distinction between coagulation and excision of the dural attachment is less reproducible than on the convexity. Consistent with this, Simpson grade 4 was the most frequent assignment in our cohort despite a median volumetric extent of resection of 96.2%. Simpson grade should, therefore, be interpreted as a descriptor of how the dural attachment was managed within this corridor rather than as a directly transferable measure of resection completeness. Finally, although the median follow-up of 65.5 months provides meaningful intermediate-term data on tumor control, longer surveillance remains necessary to fully characterize late recurrence and regrowth, particularly among patients who underwent subtotal resection or harbor residual dural disease.

Despite these limitations, the present series may serve as a reference point for future multi-institutional investigations.

## 5. Conclusions

In this single-institution series of PPFMs treated exclusively through the TL approach, favorable FN and functional outcomes were observed, with acceptable tumor control under multimodal management. In exploratory analyses, anterior petrosal zone involvement was associated with a lower likelihood of Simpson grade 1–2 resection and tumor burden with postoperative morbidity, suggesting that completeness of resection and perioperative morbidity may warrant separate consideration during operative planning; given the small cohort, these associations should be regarded as hypothesis-generating. To our knowledge, this is the first dedicated characterization of the TL approach as the sole surgical strategy in a homogeneous PPFM cohort. Because the cohort was selected on audiological and anatomical grounds, these findings describe the outcomes achievable through the TL corridor in such patients and do not establish its advantage over alternative approaches.

## Figures and Tables

**Table 1:** Baseline demographics, clinical presentation, and tumor characteristics (N = 14).

Variable	Value
***Demographics***	
Age (yr), median [IQR]	61.0 (52.5–68.0)
Female sex, *n* (%)	8 (57.1%)
BMI (kg/m^2^), median [IQR]	31.3 (27.2–38.5)
Recurrent tumor, *n* (%)	1 (7.1%)
Duration of symptoms (mos), median [IQR]	24.0 (3.7–36.0)
Clinical worsening within 30 days, *n* (%)	8 (57.1%)
***Presenting Symptoms***	
Hearing loss	10 (71.4%)
Dizziness	9 (64.3%)
Gait imbalance	8 (57.1%)
Tinnitus	5 (35.7%)
Dysphagia	4 (28.6%)
Facial numbness	4 (28.6%)
Headache	4 (28.6%)
Facial pain	3 (21.4%)
Facial weakness	2 (14.3%)
Nausea/vomiting	2 (14.3%)
Blurred vision	1 (7.1%)
Diplopia	1 (7.1%)
***Tumor Characteristics***	
Max tumor diameter (mm), median [IQR]	33.5 (27.5–42.0)
Max tumor diameter (mm), range	20–46
Preop tumor volume (cm^3^), median [IQR]	8.5 (6.3–19.7)
Preop tumor volume (cm^3^), range	3.0–30.7
Tumor laterality (left/right), *n* (%)	7 (50%)/7 (50%)
IAC extension, *n* (%)	8 (57.1%)
Cystic component, *n* (%)	0 (0%)
Petrosal surface location, *n* (%)	
Anterior only	2 (14.3%)
Middle only	1 (7.1%)
Posterior only	1 (7.1%)
Anterior + middle	2 (14.3%)
Middle + posterior	3 (21.4%)
Anterior + middle + posterior	5 (35.7%)
WHO grade, *n* (%)	
Grade I	11 (78.6%)
Grade II	3 (21.4%)
Grade III	0 (0%)
Histological subtype, *n* (%)	
Transitional	7 (50.0%)
Meningothelial	3 (21.4%)
Secretory	2 (14.3%)
Atypical	1 (7.1%)
Chordoid	1 (7.1%)
***Preoperative Status***	
Preop hydrocephalus, *n* (%)	3 (21.4%)
Residual hearing, *n* (%)	4 (28.6%)
Facial nerve function (HB), *n* (%)	
HB I	11 (78.6%)
HB II	1 (7.1%)
HB III	1 (7.1%)
HB VI	1 (7.1%)
HB I–II	12 (85.7%)
HB III–VI	2 (14.3%)

Values are *n* (%) or median [IQR]. BMI = body mass index; HB = House–Brackmann; IAC = internal auditory canal; IQR = interquartile range; mos = months. Petrosal surface location was categorized into mutually exclusive composite patterns based on the involved zones of dural attachment. For statistical analyses, anterior, middle, and posterior zone involvement was additionally coded as separate, non-mutually exclusive binary variables. Rows in bold italics are section headings that group related variables and contain no data.

**Table 2:** Surgical parameters, extent of resection, and postoperative outcomes (N = 14).

Variable	Value
***Surgical Parameters***	
Surgical approach, *n* (%)	Translabyrinthine, 14 (100%)
Surgery duration (min), median [IQR]	576.5 (501–713)
Lumbar drain placement, *n* (%)	7 (50.0%)
EVD placement, *n* (%)	2 (14.3%)
***Extent of Resection***	
EOR (%), median [IQR]	96.2 (78.8–100.0)
GTR, *n* (%)	6 (42.9%)
NTR, *n* (%)	6 (42.9%)
STR, *n* (%)	2 (14.3%)
Simpson grade, *n* (%)	
Grade 1	4 (28.6%)
Grade 2	1 (7.1%)
Grade 3	1 (7.1%)
Grade 4	8 (57.1%)
Postop tumor volume (cm^3^), median [IQR]	0.3 (0–2.1)
MRI findings, *n* (%)	
Cerebral edema	2 (14.3%)
Gliosis	1 (7.1%)
Infarction	1 (7.1%)
***Postoperative Facial Nerve Function—All Patients (n = 14)***	
At discharge—HB I–II, *n* (%)	10 (71.4%)
At 3 months—HB I–II, *n* (%)	12 (85.7%)
At 1 year—HB I–II, *n* (%)	13 (92.9%)
***Postoperative Facial Nerve Function—Preoperative HB I–II Subgroup (n = 12)***	
At discharge, *n* (%)	9 (75.0%)
At 3 months, *n* (%)	11 (91.7%)
At 1 year/last follow-up, *n* (%)	12 (100%)
***Functional Outcomes and Tumor Control***	
mRS 0–2 at discharge, *n* (%)	13 (92.9%)
mRS 0–2 at 1 year, *n* (%)	14 (100%)
GOS 4–5 at discharge, *n* (%)	12 (85.7%)
GOS 4–5 at 1 year, *n* (%)	14 (100%)
Hospital stays (days), median [IQR]	4.5 (4.0–7.5)
Discharge to home, *n* (%)	10 (71.4%)
Adjuvant radiation therapy, *n* (%)	5 (35.7%)
Tumor recurrence, *n* (%)	1 (7.1%)
Tumor regrowth, *n* (%)	3 (21.4%)
Last follow-up duration (mos), median [IQR]	65.5 (23.7–112.7)

Values are *n* (%) or median [IQR]. EOR = extent of resection; EVD = external ventricular drain; GOS = Glasgow Outcome Scale (favorable = 4–5); GTR = gross total resection; HB = House–Brackmann; IQR = interquartile range; min = minutes; mos = months; mRS = modified Rankin Scale (favorable = 0–2); NTR = near-total resection; STR = subtotal resection. Facial nerve outcomes reported as HB I–II rates; HB III–VI rates are the complement. Preoperative HB I–II subgroup (*n* = 12): all 12 patients (100%) maintained HB I–II at 1 year/last follow-up. Wilcoxon signed-rank test: discharge vs. 1-year HB, *p* = 0.083; preoperative vs. 1-year mRS, *p* = 0.317; GOS, *p* = 0.157. Rows in bold italics are section headings that group related variables and contain no data.

**Table 3:** Exploratory associations with binary extent of resection and Simpson grade.

Variable	Group 1	Group 2	*p*-Value
***Binary Extent of Resection—GTR + NTR (n = 12) vs. STR (n = 2)***			
**Categorical Variables**	**GTR + NTR (*n* = 12)**	**STR (*n* = 2)**	*p*-Value *
Anterior petrosal zone, *n* (%)	7 (58.3%)	2 (100%)	0.505
Middle petrosal zone, *n* (%)	10 (83.3%)	1 (50.0%)	1.000
Posterior petrosal zone, *n* (%)	8 (66.7%)	1 (50.0%)	1.000
IAC extension, *n* (%)	6 (50.0%)	2 (100%)	0.473
Preop hydrocephalus, *n* (%)	3 (25.0%)	0 (0%)	1.000
Recurrent tumor, *n (%)*	0 (0%)	1 (50.0%)	0.143
**Continuous Variables**	**GTR + NTR Median [IQR]**	**STR Median [IQR]**	*p*-Value *
Max tumor diameter (mm)	34.5 (28–42)	31.0 (27–35)	0.791
Preop tumor volume (cm^3^)	9.1 (6–20)	7.2 (6–10)	0.923
***Univariate Logistic Regression—Binary Extent of Resection***			
**Variable**	**OR (Exp(B))**	**95% CI**	***p*-Value**
Anterior petrosal zone	NC ‡	—	0.999
Middle petrosal zone	5.000	0.212–117.894	0.318
Posterior petrosal zone	2.000	0.098–41.003	0.653
IAC extension	NC ‡	—	0.999
Max tumor diameter (mm)	1.033	0.859–1.244	0.729
Recurrent tumor	NC ‡	—	1.000
Preop hydrocephalus	NC ‡	—	0.999
***Simpson Grade—Grade 1–2 (n = 5) vs. Grade 3–4 (n = 9)***			
**Categorical Variables**	**Simpson 1–2 (n = 5)**	**Simpson 3–4 (n = 9)**	*p*-Value *
Anterior petrosal zone, *n* (%)	1 (20.0%)	8 (88.9%)	**0.023 †**
Middle petrosal zone, *n* (%)	4 (80.0%)	7 (77.8%)	1.000
Posterior petrosal zone, *n* (%)	5 (100%)	4 (44.4%)	0.086
IAC extension, *n* (%)	4 (80.0%)	4 (44.4%)	0.301
Preop hydrocephalus, *n* (%)	0 (0%)	3 (33.3%)	0.258
Recurrent tumor, *n* (%)	0 (0%)	1 (11.1%)	1.000
**Continuous Variables**	**Simpson 1–2 Median [IQR]**	**Simpson 3–4 Median [IQR]**	*p*-Value *
Max tumor diameter (mm)	31.0 (27–38)	36.0 (29–44)	0.364
Preop tumor volume (cm^3^)	7.5 (6–12)	12.8 (7–21)	0.518
***Univariate Logistic Regression—Simpson Grade***			
**Variable**	**OR (Exp(B))**	**95% CI**	***p*-Value**
Anterior petrosal zone	0.031	0.002–0.641	**0.025 †**
Posterior petrosal zone	NC ‡	—	0.999
Middle petrosal zone	1.143	0.077–16.947	0.923
IAC extension	5.000	0.388–64.387	0.217
Max tumor diameter (mm)	0.925	0.799–1.070	0.295
Preop hydrocephalus	NC ‡	—	0.999
Recurrent tumor	NC ‡	—	1.000

Values are n (%) or median [IQR]. CI = confidence interval; GTR = gross total resection; IAC = internal auditory canal; IQR = interquartile range; Max = maximum; NTR = near-total resection; OR = odds ratio; Preop = preoperative; STR = subtotal resection. * Fisher’s exact test for categorical variables; Mann–Whitney U test for continuous variables. † Statistically significant (*p* < 0.05). Anterior petrosal zone involvement was the only factor significantly associated with Simpson grade, corresponding to lower odds of Simpson grade 1–2 resection (an OR < 1 indicates lower odds of grade 1–2 and, conversely, higher odds of grade 3–4). It was not significantly associated with volumetric extent of resection. ‡ NC = not computable; complete separation due to small sample size (OR approaches 0 or infinity). Rows in bold italics are section headings that group related variables and contain no data; data rows in bold denote statistically significant associations.

**Table 4:** Postoperative complications (N = 14).

Variable	*n* (%)
***Complications***	
Any complication	8 (57.1%)
Postoperative shunt placement	4 (28.6%)
Cranial nerve VI palsy	2 (14.3%)
Postoperative dysphagia requiring swallowing therapy	3 (21.4%)
CSF leak	2 (14.3%)
Reoperation for wound complication	1 (7.1%)
Postoperative hematoma	0 (0%)
Rhinorrhea	0 (0%)
Otorrhea	0 (0%)
Facial dyskinesia	0 (0%)
Medical complications	0 (0%)

Values are *n* (%). “Any complication” refers to patients with at least one postoperative complication; because some patients had multiple complications, individual complication categories do not sum to the total number of patients with any complication. Rows in bold italics are section headings that group related variables and contain no data. CSF = cerebrospinal fluid.

**Table 5:** Exploratory univariate analyses of postoperative complications and perioperative outcomes.

Variable	No Complication (*n* = 6)	Any Complication (*n* = 8)	*p*-Value *
***Categorical Variables—Fisher’s Exact Test***			
Anterior petrosal zone, *n* (%)	3 (50.0%)	6 (75.0%)	0.580
Middle petrosal zone, *n* (%)	6 (100%)	5 (62.5%)	0.209
Posterior petrosal zone, *n* (%)	5 (83.3%)	4 (50.0%)	0.301
IAC extension, *n* (%)	5 (83.3%)	3 (37.5%)	0.138
Preop hydrocephalus, *n* (%)	0 (0%)	3 (37.5%)	0.209
Recurrent tumor, *n* (%)	0 (0%)	1 (12.5%)	1.000
***Continuous Variables—Median [IQR]***			
**Variable**	**No Complication Median [IQR]**	**Any Complication Median [IQR]**	*p*-Value *
Max tumor diameter (mm)	30 (27–34)	40 (33–45)	0.081
Preop tumor volume (cm^3^)	7.1 (6–10)	14.8 (8–22)	0.228
Surgery duration (min)	540 (490–600)	610 (530–740)	0.282
***Significant Exploratory Associations***			
**Variable**	**Statistic**	**Value**	***p*-Value**
Tumor diameter vs. complication count	Spearman ρ	0.585	**0.028 †**
Hydrocephalus vs. complication count	MWU	11.83 vs. 6.32	**0.030 †**
Tumor volume vs. surgery duration	Spearman ρ	0.607	**0.021 †**
Tumor volume vs. hospital stays	Spearman ρ	0.753	**0.002 †**
Tumor volume vs. CSF leak	MWU	U = 0.000	**0.022 †**
***Univariate Logistic Regression—Binary Complication Outcome***			
**Variable**	**OR (Exp(B))**	**95% CI**	***p*-Value**
Anterior petrosal zone	3.000	0.312–28.841	0.341
Middle petrosal zone	NC ‡	—	0.999
Posterior petrosal zone	0.200	0.016–2.575	0.217
IAC extension	0.120	0.009–1.584	0.107
Max tumor diameter (mm)	1.145	0.969–1.352	0.112
Preop tumor volume (cm^3^)	1.000	1.000–1.000	0.126
Surgery duration (min)	1.005	0.997–1.014	0.231
Recurrent tumor	NC ‡	—	1.000
Preop hydrocephalus	NC ‡	—	0.999

Values are *n* (%) or median [IQR]. CSF = cerebrospinal fluid; IAC = internal auditory canal; IQR = interquartile range; MWU = Mann–Whitney U test; ρ = Spearman rank correlation coefficient. * Fisher’s exact test for categorical variables; Mann–Whitney U test for continuous variables. † Statistically significant (*p* < 0.05). No variable reached significance for any complication as a binary outcome (all *p* > 0.05; see univariate logistic regression section above). In exploratory analyses, associations were identified for complication count as a continuous outcome and for specific complications (CSF leak, surgery duration, hospital stay). Rows in bold italics are section headings that group related variables and contain no data; data rows in bold denote statistically significant associations. ‡ NC = not computable; complete separation due to small sample size (OR approaches 0 or infinity).

**Table 6:** Anatomical classification of petrous region meningiomas: surgical approach selection and role of the translabyrinthine approach.

Subtype	Dural Origin/ Location	Key Anatomical Relations	Surgical Approach(es)	Role of TL Approach—Indications and Deviation Factors *
***Posterior Petrous Face Meningiomas—Primary Indication for the Translabyrinthine Approach***				
Posterior Petrous Face—Premeatal (Anterior to IAC)	Posterior petrous surface, anterior to IAC	Lateral to CN V; CN VII–VIII anterio- medially displaced; IAC frequently involved; may mimic petroclival extension	TL (preferred: IAC involvement or non-serviceable hearing) RS (hearing preservation; minimal IAC involvement)	Approach: RS when hearing is serviceable and IAC is not involved; TL when hearing is non-serviceable or IAC/fundal involvement. TL indicated: Non-serviceable hearing (PTA > 50 dB or WRS < 50%); IAC/fundal involvement; large tumor (≥3–4 cm) with obliterated CPA cistern; anterior-medial growth vector; unfavorable venous anatomy (high jugular bulb, dominant petrosal vein); absence of arachnoid plane between tumor and CN VII–VIII; prior surgery or radiation with scarred CPA planes. Note: TL allows early FN identification at fundus before tumor dissection; direct IAC access without cerebellar retraction.
Posterior Petrous Face—Meatal (Centered on IAC)	Posterior petrous surface, centered on IAC	Lateral to CN V; Tumor centered on IAC; CN VII–VIII at highest risk; fundal involvement nearly universal	TL (preferred; IAC involvement inherent) RS + RL (only if hearing serviceable and fundus spared; no anterior extension)	Approach: TL is the default—IAC involvement is inherent in this subtype; RS + retrolabyrinthine only when hearing serviceable, fundus spared, and no anterior extension. TL indicated: IAC/fundal involvement; non-serviceable hearing; anterior extension requiring ETLA with transapical corridor; absence of arachnoid plane between tumor and CN VII–VIII; fibrous tumor consistency with dense nerve adhesion. Note: Direct lateral-to-medial IAC access enables FN identification at fundus prior to any dissection—critical when arachnoid plane is absent.
Posterior Petrous Face—Retromeatal (Posterior to IAC)	Posterior petrous surface, posterior to IAC; pure CPA	Lateral to CN V; posterior to IAC; CN VII–VIII displaced anteriorly; favorable CPA access	RS (preferred; hearing preservation possible) RS + RL (hearing preservation with wider exposure) TL (non-serviceable hearing or large tumor)	Approach: RS or RS + retrolabyrinthine preferred—favorable posterior CPA location allows hearing preservation; TL when hearing is non-serviceable or approach anatomy unfavorable. TL indicated: Non-serviceable or absent hearing; large tumor (≥3–4 cm) obliterating CPA corridor; high-riding jugular bulb restricting RS dissection; prior surgery or radiation with obliterated arachnoid planes; absence of tumor-nerve arachnoid plane (en plaque or invasive variants). Note: RS retained when serviceable hearing; broad arachnoid planes; favorable FN displacement.
***Subtypes Requiring Additional or Combined Approaches—Translabyrinthine Approach Alone Insufficient***				
Anterior Petrous (Premeckelian)	Anterior petrous surface, anterior to IAC; middle cranial fossa	Anterior/lateral to CN V; near Meckel’s cave and Gasserian ganglion; vein of Labbé at risk	Kawase (anterior petrosectomy) Subtemporal Extended middle fossa ±TL if posterior CPA extension	Approach: Kawase or extended middle fossa—upward anterior trajectory not achievable via TL alone; TL not applicable as sole approach. TL indicated: Combined TL + Kawase when posterior CPA extension coexists with anterior petrous involvement; TL provides posterior corridor while Kawase addresses anterior component. Note: Hearing often serviceable → hearing preservation prioritized; anterior approach selected accordingly.
Petroclival	Medial petrous bone and upper clivus (petroclival junction); medial to CN V	MEDIAL to CN V (key distinguisher); displaces CN V posterolaterally; near basilar artery, brainstem, CN VI at Dorello’s canal	Presigmoid ± posterior petrosectomy Kawase Combined transpetrosal RS (select cases)	Approach: Combined transpetrosal or presigmoid; medial location requires multi-corridor access; RS for smaller tumors with limited anterior extension and serviceable hearing. TL indicated: Posterior component of combined approach when posterior CPA involvement is significant and hearing is non-serviceable; TL alone is insufficient for clival or medial extension. Note: Tumor medial to CN V (displaces CN V posterolaterally)—key anatomical distinguisher from posterior petrous face meningioma.
Petrous Apex	Deep petrous apex (not a surface tumor); adjacent to Meckel’s cave	Medial/anterior to IAC; adjacent to CN V and Meckel’s cave; near petrous ICA and CN VI	Kawase Extended middle fossa RS Endonasal (select cases)	Approach: Kawase or extended middle fossa for anterior/deep access; RS when posterior CPA extension is the dominant component. TL indicated: Combined TL + Kawase when posterior petrous surface and deep apex both involved and hearing non-serviceable; TL alone does not reach the deep petrous apex. Note: Petrous ICA proximity mandates preoperative vascular imaging; hearing often functional.
Petrotentorial (Tentorial–Petrous Junction)	Petrous ridge and tentorial junction; may span both fossae	Variable medial/ lateral to IAC; involves tentorium and CN IV; superior petrosal sinus at risk	Combined subtemporal–RS Supracerebellar infratentorial Transtentorial ±TL if posterior component	Approach: Combined subtemporal–RS or supracerebellar infratentorial with transtentorial extension; RS provides standard infratentorial access. TL indicated: Replaces or augments RS component when posterior petrous surface involved and hearing non-serviceable; provides wider lateral CPA exposure for combined supra/infratentorial corridor. Note: Superior petrosal sinus and CN IV management critical regardless of approach.
Petro-Occipital (Posteromedial Petrous)	Posterior petrous surface near jugular foramen; petro-occipital junction	Posteroinferior to IAC; near jugular bulb; CN IX–XI at risk; vertebral artery proximity	RS Far-lateral Transcondylar (select cases) ±TL if labyrinthine involvement	Approach: RS for superior component; far-lateral for inferior or condylar extension; combined as needed. TL indicated: Combined with RS when posterior petrous and labyrinthine involvement coexist and hearing is non-serviceable; high-riding jugular bulb is a relative contraindication to TL (drilling hazard). Note: CN IX–XI monitoring and jugular bulb anatomy determine TL feasibility.
Intrapetrous (Intraosseous)	Within petrous bone; no clear dural surface origin; may involve ICA canal, cochlea, or IAC	Variable—depends on intraosseous expansion pattern; may erode ICA canal or cochlea	Tailored: TL Kawase Endonasal (select) Combined	Approach: Imaging-guided tailored approach; may mimic schwannoma or cholesteatoma; TL, Kawase, endonasal, or combined depending on expansion pattern. TL indicated: Posterior petrous/labyrinthine involvement with non-serviceable hearing; Kawase or combined when ICA canal or anterior petrous apex involved. Note: ICA canal proximity mandates preoperative vascular imaging.

CN = cranial nerve; CPA = cerebellopontine angle; IAC = internal auditory canal; ICA = internal carotid artery; PTA = pure-tone average; RS = retrosigmoid; TL = translabyrinthine; WRS = word recognition score. * Surgical approach selection is multifactorial. Hearing status alone does not determine approach. Decision factors include: (1) preoperative hearing function (PTA, WRS); (2) IAC and fundal anatomy; (3) tumor volume and growth vector; (4) facial nerve course and displacement pattern; (5) venous and vascular anatomy; (6) presence or absence of arachnoid plane between tumor and CN VII–VIII; (7) prior surgery or radiation; and (8) extent of tumor beyond the posterior petrous surface. Petroclival meningiomas are anatomically distinct from posterior petrous face meningiomas. The key distinguishing feature is the relationship to CN V: petroclival tumors arise medial to CN V and displace it posterolaterally; posterior petrous meningiomas arise lateral to CN V and displace CN VII–VIII anteromedially. For pure posterior petrous face meningiomas (premeatal, meatal, retromeatal subtypes), the translabyrinthine approach may be sufficient in appropriately selected patients. Extension to the anterior petrous surface, clivus, petrous apex, tentorium, or petro-occipital region requires additional or combined surgical corridors. While the present institutional cohort comprised exclusively posterior petrous face meningiomas, the broader anatomical framework is included to contextualize the role of the translabyrinthine corridor within the spectrum of petrous region meningiomas and to delineate the boundaries beyond which alternative or combined approaches become necessary. Classification adapted from published surgical frameworks [1,4,5,9,10,11,13,15]. The approach selection principles and deviation factors listed in this table are derived from those published frameworks and from institutional practice; they were not derived from, nor validated by, the outcomes of the present cohort, which comprised posterior petrous face meningiomas only. Entries for other petrous region entities are provided for anatomical context and represent literature-based and institutional recommendations rather than findings of this study. Rows in bold italics are section headings that group tumor subtypes and contain no data.

## Data Availability

The data that support the findings of this study are available from the corresponding author upon reasonable request.

## Abbreviations

The following abbreviations are used in this manuscript:

CI	Confidence interval
CPA	Cerebellopontine angle
CSF	Cerebrospinal fluid
EOR	Extent of resection
EVD	External ventricular drain
FN	Facial nerve
GOS	Glasgow Outcome Scale
GTR	Gross total resection
HB	House–Brackmann
IAC	Internal auditory canal
IQR	Interquartile range
LD	Lumbar drain
MRI	Magnetic resonance imaging
mRS	Modified Rankin Scale
NTR	Near-total resection
OR	Odds ratio
PPFM	Posterior petrous face meningioma
STR	Subtotal resection
TL	Translabyrinthine
